# Comparative Study of Ti- and Cu-Doped Zn_2_SnO_4_ Films Deposited by Spray Pyrolysis for UV Photodetection

**DOI:** 10.3390/s26196321

**Published:** 2026-10-07

**Authors:** Vadim Morari, Cristian Casut, Daniel Ursu, Eduard V. Monaico, Emil V. Rusu, Marinela Miclau, Veaceslav V. Ursaki

**Affiliations:** 1 Institute of Electronic Engineering and Nanotechnologies “D. Ghitu”, Technical University of Moldova, Academiei Str. 3/3, MD-2028 Chisinau, Moldova; emil.rusu@iien.utm.md; 2 National Institute for Research and Development in Electrochemistry and Condensed Matter, 300569 Timişoara, Romania; cristian.casut95@e-uvt.ro (C.C.); danielhoratiu@yahoo.com (D.U.); marinela.miclau@gmail.com (M.M.); 3 National Center for Materials Study and Testing, Technical University of Moldova, Bv. Stefan cel Mare 168, MD-2004 Chisinau, Moldova; eduard.monaico@cnstm.utm.md; 4 Academy of Sciences of Moldova, Bv. Stefan cel Mare 1, MD-2001 Chisinau, Moldova; vvursaki@gmail.com

**Keywords:** Zn_2_SnO_4_, spray pyrolysis, oxide films, Ti doping, Cu doping, UV photodetector, responsivity, detectivity

## Abstract

This study aims to clarify how Ti and Cu doping differently influence the structural, morphological, electrical, and UV photodetection properties of Zn_2_SnO_4_ films prepared by spray pyrolysis. The comparison is based on the different chemical and electronic characteristics of the dopants and their resulting effects on charge transport and photoresponse. Zn_2_SnO_4_ films doped with 1–3 vol.% Ti or Cu were deposited under identical conditions and characterized using X-ray diffraction, atomic force microscopy, electrical measurements, and transient UV photoresponse at 365 nm. The results demonstrate distinct dopant-dependent structure–property relationships. Ti doping maintains a relatively homogeneous surface morphology and results in higher sheet resistance, whereas Cu doping produces stronger concentration-dependent morphological changes and lower sheet resistance at 1–2 vol.% Cu. The different electrical characteristics are reflected in the UV photodetection performance. The 1 vol.% Ti-doped film exhibits the highest responsivity of 239 mA/W, specific detectivity of 2.73 × 10^10^ Jones and a recovery time (τ_90_) of 74 ms, while the 3 vol.% Cu-doped film achieves the highest photo-to-dark current ratio of approximately 763 and a fast recovery time (τ_90_) of 70 ms. These results demonstrate that Ti and Cu provide different routes for tailoring Zn_2_SnO_4_ films for UV photodetection: Ti doping favors high responsivity and detectivity, whereas Cu doping enables efficient dark current suppression and rapid photoresponse. This study therefore establishes a direct relationship between dopant-dependent material properties and UV photodetector performance.

## 1. Introduction

In recent years, increasing research efforts have been devoted to oxide films, ternary oxide semiconductors, nanostructured oxide materials, and oxide-based heterostructures due to their remarkable structural, optical, electrical, and photoelectrical properties, which make them highly attractive for a wide range of technological and optoelectronic applications. Among ternary oxide semiconductors, Zn_2_SnO_4_ has attracted considerable attention due to its wide band gap, high optical transparency in the visible region, low electrical resistivity, remarkable chemical stability, and excellent photoelectrical properties, making it a promising material for transparent electronics, UV photodetectors, solar cells, gas sensors, and various optoelectronic devices [1,2,3,4,5,6,7]. Zn_2_SnO_4_ belongs to the family of transparent conducting oxides and crystallizes in a cubic inverse spinel structure [8], which contributes to its structural stability and favorable electrical transport characteristics. Its wide band gap, typically in the range of 3.3–3.7 eV depending on deposition conditions and post-treatment processes, ensures high transparency in the visible range while maintaining sensitivity in the ultraviolet region [9]. These features make Zn_2_SnO_4_ particularly attractive for applications requiring transparent and UV-responsive materials [10,11]. Among various deposition techniques, spray pyrolysis is widely used for the fabrication of oxide semiconductor films due to its simplicity, low cost, high reproducibility, and suitability for large-area deposition [12,13,14]. Compared with vacuum-based techniques such as magnetron sputtering [15], pulsed laser deposition [16], thermal evaporation [17], or atomic layer deposition [18], spray pyrolysis offers several advantages, including low equipment cost, atmospheric pressure processing, easy control of precursor composition, and the ability to deposit uniform coatings on different substrates. Consequently, it has become one of the most attractive methods for preparing transparent conducting oxides and functional semiconductor films for electronic, photovoltaic, gas sensing, and optoelectronic applications. The physical properties of Zn_2_SnO_4_ films strongly depend on the deposition parameters such as substrate temperature, precursor concentration, spray rate, annealing treatment, and substrate type. Furthermore, this technique allows for precise incorporation of dopants into the host matrix through simple modification of the precursor solution, making it particularly suitable for multicomponent oxide systems [19]. One of the most effective approaches for tailoring the structural, electrical, and optical properties of Zn_2_SnO_4_ films is doping with suitable elements. Doping can modify carrier concentration, conductivity type, defect density, grain growth, and recombination processes, thereby improving device performance. Depending on the ionic radius and valence state of the dopant, substitution at Zn or Sn lattice sites may occur, leading to changes in lattice parameters, crystallinity, and electronic structure. In addition, dopants may introduce shallow donor or acceptor levels that influence charge transport and photoelectrical behavior. For example, in amorphous Zn–Sn–O film transistors, Ti doping induces a clear mobility–stability tradeoff: increasing Ti content enhances gate-bias stability by suppressing oxygen vacancies but reduces field-effect mobility. An optimal concentration (~1.14 at. %) provides a balanced performance with high mobility and an excellent ON/OFF ratio [20]. Similarly, N-incorporated Zn_2_SnO_4_ nanostructures have been reported to exhibit band-gap narrowing and an enhanced photocurrent response, which were attributed to the formation of impurity levels and improved charge separation efficiency [21]. Further improvements in photoelectrical performance have been reported in Zn_2_SnO_4_ systems doped with transition metals such as Co and Ni, where dopant-induced energy levels extend light absorption into the visible region and enhance charge carrier separation, resulting in improved photocatalytic CO_2_ reduction [22]. In Fe- and Ca-doped Zn_2_SnO_4_ nanoparticles, compositional tuning influences both the optical band gap and antibacterial activity, with higher dopant concentrations generally enhancing functional performance through defect engineering and structural modification [23,24]. In dielectric oxide ceramics such as ZnSnO_3_, Al and Mg substitution plays a crucial role in controlling microstructure, defect density, and grain growth. Al doping leads to grain refinement, reduced structural disorder, and improved dielectric performance with low losses, while Mg incorporation enables the tuning of the band gap, dielectric constant, and charge transport behavior through polarization effects and defect-related conduction mechanisms [25,26].

Doping has emerged as an effective strategy for tailoring the structural, electronic, and functional properties of Zn_2_SnO_4_. However, studies concerning Ti and Cu incorporation into this material remain relatively limited. Ti doping has been reported to enhance the electrochemical performance of Zn_2_SnO_4_ by improving its cycling stability and lithium storage capability [27]. Cu doping has recently attracted considerable attention because it has been reported to alter the *n*-type semiconductor properties of Zn_2_SnO_4_ by improving electron extraction and suppressing electron–hole recombination, making it particularly attractive for photoelectrical and optoelectronic applications [28]. Despite these promising results, a direct comparison between Ti- and Cu-doped Zn_2_SnO_4_ films prepared under identical spray pyrolysis conditions has not yet been reported. Such a comparative study is important for understanding how different dopants influence the relationships between microstructure, electrical transport, and UV photodetection performance. The physical motivation for comparing Ti and Cu as dopants in Zn_2_SnO_4_ arises from their different chemical and electronic characteristics. In particular, Ti^4+^ is aliovalent with respect to Zn^2+^, whereas Cu^2+^ is nominally isovalent with Zn^2+^. Consequently, their incorporation into the Zn_2_SnO_4_ host can be expected to produce different effects on the local defect chemistry, carrier concentration, charge transport pathways, and carrier trapping and recombination processes. These differences provide a physical basis for investigating whether Ti and Cu modify UV photodetection primarily through changes in photocurrent generation, dark current suppression, charge transport, or carrier relaxation. Since the present study does not include complementary techniques capable of directly identifying specific dopant sites or defect states, these effects are discussed in terms of experimentally observed structure–property relationships rather than as direct evidence of a particular substitution mechanism.

A direct comparison of Ti and Cu at identical dopant concentrations and under identical deposition and measurement conditions is therefore particularly relevant. Such a controlled comparison allows the influence of dopant chemistry on the structural, morphological, electrical, and photoelectrical properties of Zn_2_SnO_4_ to be distinguished and correlated with key UV photodetection parameters, including photocurrent, photo-to-dark current ratio, responsivity, specific detectivity, and recovery time (τ_90_).

In this work, we systematically compare the effects of Ti and Cu doping on the structural, morphological, electrical, and UV photodetection properties of Zn_2_SnO_4_ films deposited by spray pyrolysis under identical experimental conditions. The physical motivation for this comparison arises from the different chemical and electronic characteristics of Ti and Cu, which can lead to different modifications of the defect chemistry and charge transport behavior of the Zn_2_SnO_4_ host. This study aims to establish the relationship between dopant type and concentration, the resulting structural and morphological characteristics, electrical transport, and UV photodetection performance. In particular, the effects of 1–3 vol.% Ti and Cu doping are evaluated in terms of crystal structure, surface morphology, current–voltage characteristics, photo-to-dark current ratio, responsivity, specific detectivity, and transient response. By correlating these properties under identical deposition and measurement conditions, this work provides a direct comparison of the different roles of Ti and Cu doping in tailoring Zn_2_SnO_4_ films for UV photodetection.

## 2. Materials and Methods

### 2.1. Materials and Film Deposition

**Figure 1:**
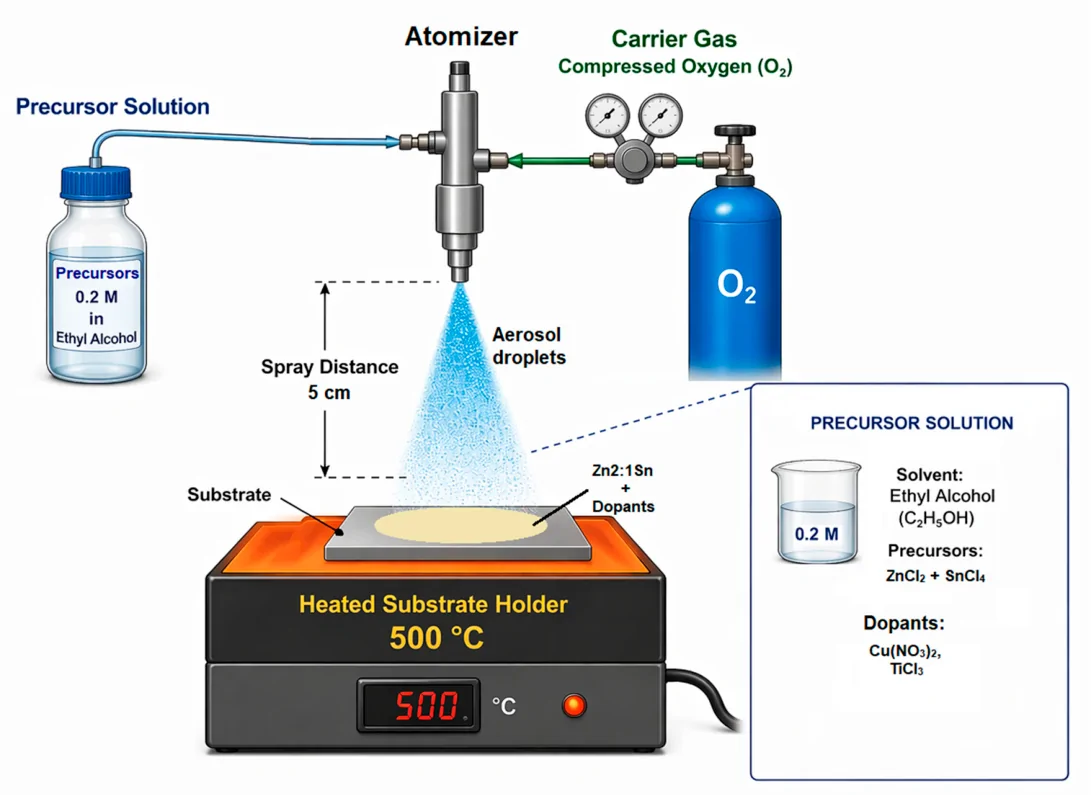
Schematic illustration of spray pyrolysis setup used for Zn-Sn-O film deposition.

Ti and Cu (1–3 vol.%)-doped Zn_2_SnO_4_ films were deposited on *n*-type silicon substrates (Si-100) by using the spray pyrolysis technique (see Figure 1). Prior to deposition, the silicon wafers were cut into pieces with dimensions of 15 mm × 15 mm and chemically cleaned to remove surface contaminants and native impurities in NH_4_OH:H_2_O_2_:H_2_O (1:1:4) for 15 min at 70 °C. Then, they were held in HNO_3_:HF (3:1) at room temperature for 2 min, followed by rinsing with distilled water. The precursor solutions were prepared using zinc chloride (ZnCl_2_) and tin tetrachloride (SnCl_4_) as Zn and Sn sources, respectively. Each salt was dissolved in ethanol at a concentration of 0.2 M. Zn_2_SnO_4_-based films were prepared using Zn molar ratios of 1:1, 2:1, and 1:2. For all compositions, the corresponding amounts of ZnCl_2_ and SnCl_4_ were mixed to obtain the desired Zn molar ratio while maintaining the same precursor concentration and deposition conditions. The films were deposited by spray pyrolysis at 500 °C for 5 min. To modify the electrical and photoelectrical properties of the deposited films, different metallic dopants were introduced into the precursor solution. Cu(NO_3_)_2_ and TiCl_3_ were used as precursor salts for Cu and Ti doping, respectively. The Ti and Cu concentrations of 1–3 vol.% refer to the nominal dopant concentrations in the precursor solutions used for spray pyrolysis. These values were calculated from the corresponding volumes of the Ti- or Cu-containing precursor relative to the precursor solution used for deposition. No independent compositional analysis, such as energy-dispersive X-ray spectroscopy (EDS) or X-ray photoelectron spectroscopy (XPS), was performed to determine the actual Ti or Cu content in the deposited films. Therefore, the reported concentrations represent nominal precursor compositions and should not be interpreted as experimentally verified Ti or Cu contents in the deposited films.

The deposition time for all samples was fixed at 5 min. During spray pyrolysis deposition, the precursor solution was atomized and transported toward a substrate heated to 500 °C with a nozzle-to-substrate distance of 5 cm. Upon contact with the heated substrate, the precursor droplets underwent rapid solvent evaporation followed by thermal decomposition and oxidation, resulting in the formation of metal-doped Zn_2_SnO_4_ films. After deposition, two coplanar aluminum electrodes with a diameter of 1 mm and an interelectrode distance of 5 mm were fabricated on the Zn_2_SnO_4_-based films by thermal evaporation under vacuum conditions (10^−5^ Torr) at 200 °C for 1 h and subsequently used for electrical and photoelectrical measurements.

### 2.2. Characterization

The surface morphology of the deposited films was characterized by atomic force microscopy (AFM) using a Surface Imaging NanoStation II system operated in non-contact mode with a silicon (Si) cantilever. The acquired AFM images were analyzed using Gwyddion 2.69 software (Czech Metrology Institute, Brno, Czech Republic) to determine the surface roughness parameters. Particle size distributions were obtained by analyzing the AFM images using ImageJ 1.54s software, from which the average particle size and corresponding histograms were determined.

The electrical and photoelectrical characteristics of the Zn_2_SnO_4_-based films were investigated under dark and UV illumination conditions at an applied bias voltage of 5 V. UV illumination was provided by a 365 nm UV LED. The incident optical power density at the sample position was measured to be 430 mW/cm^2^, corresponding to an incident optical power of approximately 0.0344 W for the effective active area of 0.08 cm^2^. A systematic investigation of the responsivity as a function of incident optical power was not performed because the primary objective of this study was to compare the photoelectrical performance of the investigated Zn_2_SnO_4_-based photodetectors under identical and reproducible illumination conditions. The dark and illuminated currents used to calculate the photo-to-dark current ratio, responsivity, and specific detectivity were obtained from the corresponding I–V characteristics at a bias voltage of 5 V. In contrast, the transient photocurrent (I–t) measurements were performed under periodic UV light ON/OFF switching and were used to evaluate the recovery times (τ_90_). Structural analysis of the samples was carried out using an X’Pert PRO MPD X-ray diffractometer (Philips—FEI Company PANalytical B.V., Almeo, The Netherlands, 2007), equipped with a copper (Cu) anode X-ray tube and a PixCEL detector. The system features a vertical θ–θ goniometer and a rotating sample stage with programmable rotation speed. The high-voltage generator operates at a maximum voltage of 60 kV and a maximum current of 55 mA. Data acquisition and goniometer control are performed using dedicated software, which enables the recording of X-ray diffraction patterns in both reflection and transmission geometries.

The electrical resistivity of the films was measured using a Jandel four-point probe resistivity measurement system (RM3000, Jandel Engineering Ltd., Leighton Buzzard, UK) operating in the standard four-point probe configuration. The technique eliminates the influence of contact resistance, providing accurate measurements of sheet resistance and electrical resistivity of the deposited films.

## 3. Results and Discussion

**Figure 2:**
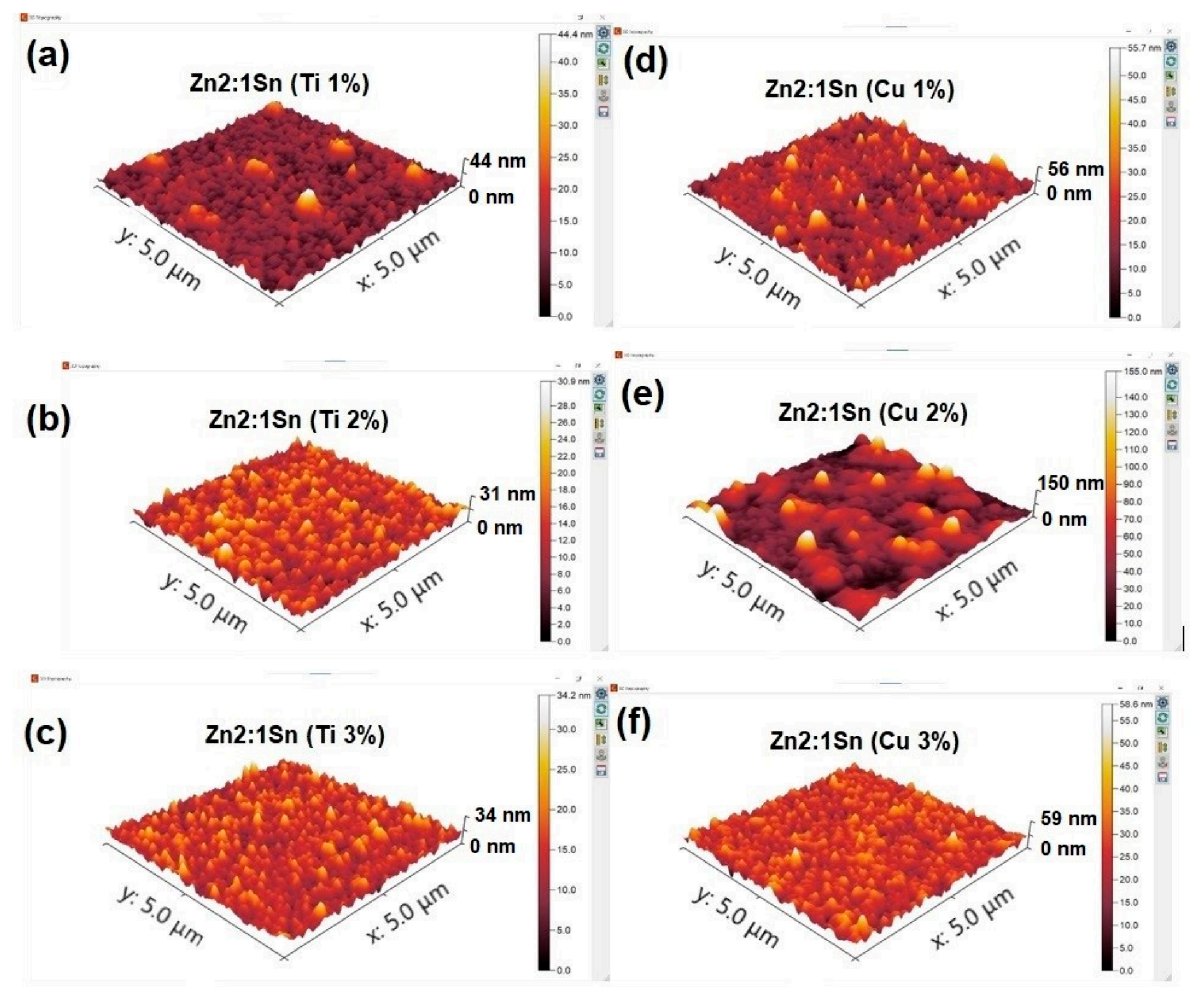
Three-dimensional AFM surface topography images of Zn2:1Sn oxide films doped with (**a**) Ti 1 vol.%, (**b**) Ti 2 vol.%, (**c**) Ti 3 vol.%, (**d**) Cu 1 vol.%, (**e**) Cu 2 vol.%, and (**f**) Cu 3 vol.%. All images were acquired over a 5 × 5 μm^2^ scan area.

The surface topography of the deposited Zn2:1Sn oxide films doped with Ti (1–3 vol.%) and Cu (1–3 vol.%) was investigated by atomic force microscopy (AFM). Figure 2 presents the three-dimensional AFM surface topographies acquired over a scan area of 5 × 5 μm^2^. The Ti-doped films exhibit a compact and homogeneous granular morphology with uniformly distributed nanograins. The film containing 1 vol.% Ti (Figure 2a) shows a relatively smooth surface with a maximum height of approximately 44 nm. Increasing the Ti concentration to 2 vol.% (Figure 2b) results in a more densely packed morphology and a reduced height variation of about 31 nm, while the surface remains highly uniform. At 3 vol.% Ti (Figure 2c), the maximum height slightly increases to approximately 34 nm without significant changes in the overall morphology. These observations are supported by the roughness analysis, where the root mean square roughness (Sq) changes only slightly from 4.425 nm to 4.287 nm and 5.130 nm, while the arithmetic mean roughness (Sa) varies from 3.164 nm to 3.386 nm and 4.125 nm for 1, 2, and 3 vol.% Ti, respectively. The small variations in both Sa and Sq indicate that the surface morphology remains relatively stable over the investigated Ti concentration range. The limited variation in surface roughness suggests that the nominal Ti concentration is associated with only minor changes in the observed surface topography.

In contrast, Cu doping has a more pronounced impact on the surface topography. The Zn2:1Sn film doped with 1 vol.% Cu (Figure 2d) exhibits a granular morphology with randomly distributed protrusions and a maximum height of approximately 56 nm. A pronounced morphological transformation occurs at 2 vol.% Cu (Figure 2e), where large grain agglomerates and hill-like features dominate the surface, increasing the maximum height to approximately 150 nm. Correspondingly, the roughness parameters increase dramatically, with Sq rising from 6.051 nm to 21.56 nm and Sa from 4.511 nm to 16.24 nm, indicating a considerably rougher surface with more pronounced agglomerated surface features. When the Cu concentration is further increased to 3 vol.% (Figure 2f), the surface becomes finer and more homogeneous, accompanied by a decrease in the maximum height to approximately 59 nm. Accordingly, the roughness values decrease to Sq = 6.022 nm and Sa = 4.767 nm, approaching those of the 1 vol.% Cu-doped film.

Overall, the AFM analysis demonstrates that Ti doping is associated with relatively small changes in surface morphology over the investigated concentration range, whereas Cu doping exhibits a more pronounced and non-monotonic morphological evolution. In particular, the 2 vol.% Cu-doped film exhibits the highest surface roughness and the most pronounced agglomerated surface features, while increasing the Cu content to 3 vol.% is associated with a decrease in roughness and a more uniform surface morphology. The origin of this non-monotonic behavior cannot be established from AFM measurements alone and may depend on several factors related to film formation and the nominal dopant concentration.

The observed morphological variations may affect the density and distribution of grain boundaries and surface features, which could in turn influence charge transport and UV photodetection behavior, as discussed in the following sections. However, these relationships should be considered possible correlations rather than direct evidence of a causal mechanism.

**Figure 3:**
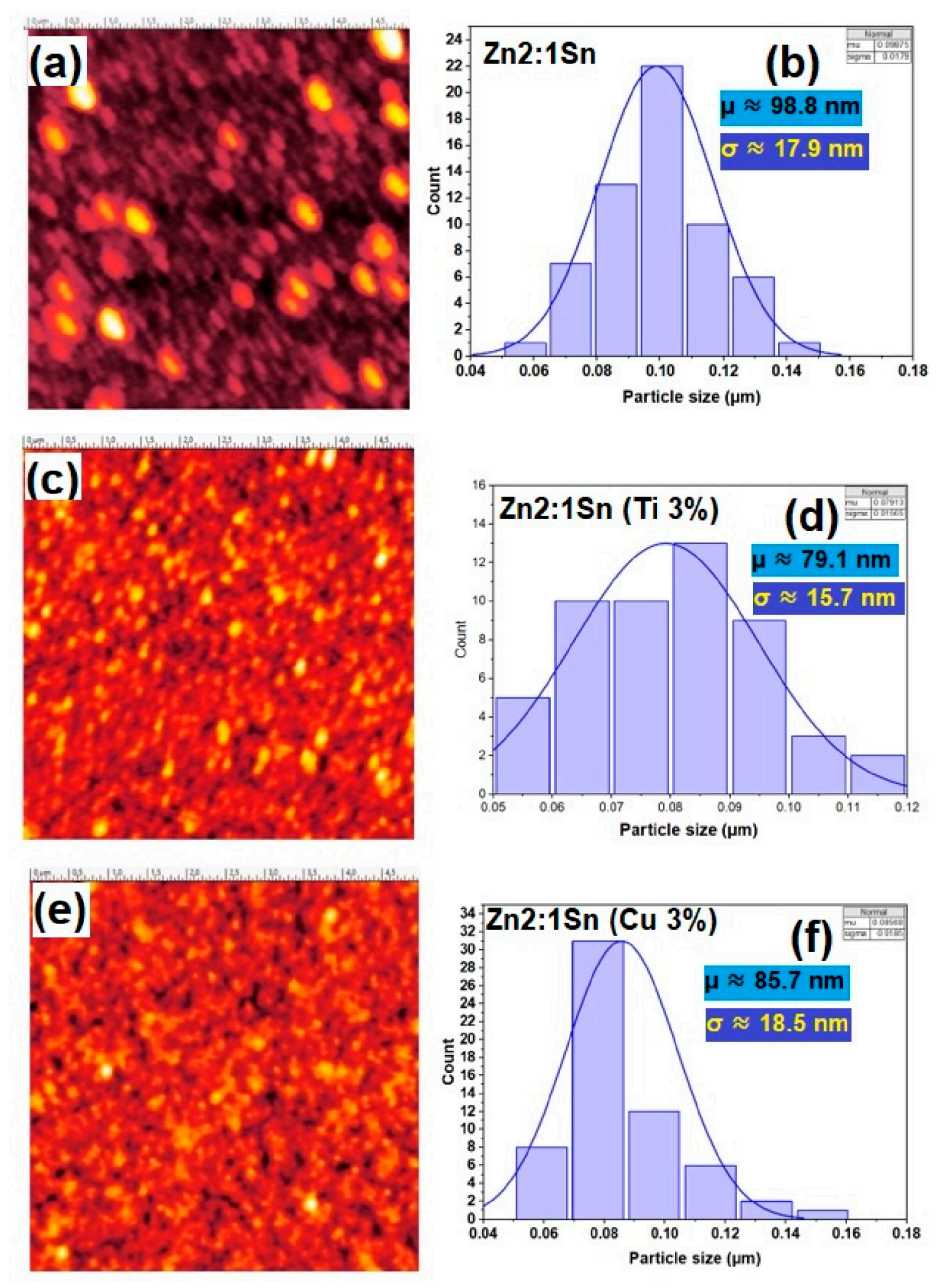
Representative AFM images and corresponding particle size distribution histograms of (**a**,**b**) undoped Zn2:1Sn, (**c**,**d**) Zn2:1Sn (Ti 3 vol.%), and (**e**,**f**) Zn2:1Sn (Cu 3 vol.%). The histograms were fitted with a Gaussian distribution to determine the mean grain size (μ) and standard deviation (σ).

To further quantify the influence of dopant incorporation on the microstructure, the particle size distribution was determined from the AFM images, and the corresponding results are presented in Figure 3.

Representative AFM images together with the particle size histograms reveal that both Ti and Cu doping modify the average grain size and its distribution. The undoped Zn2:1Sn oxide film exhibits an average particle size of approximately 98.8 ± 17.9 nm. The Ti-doped film exhibits a slightly narrower particle size distribution together with a reduced average particle size, indicating a more homogeneous surface morphology. In contrast, the Zn2:1Sn (Cu 3%) oxide film exhibits an intermediate average particle size of 85.7 ± 18.5 nm, which is lower than that of the undoped film. The AFM statistical analysis indicates that the mean particle size depends on both dopant type and concentration. Ti doping generally results in smaller mean particle sizes than the undoped film, whereas Cu doping produces a non-monotonic variation, with a pronounced increase at 2 vol.% Cu. The particle size distributions remain predominantly unimodal, without distinct secondary populations of surface features. To provide a comprehensive overview of the surface characteristics, all roughness parameters and grain size distributions for all Ti and Cu concentrations, as well as for the undoped films, are summarized in Table 1.

A limitation of the present morphological investigation is the absence of SEM characterization. Although AFM provides quantitative information on the three-dimensional surface topography and roughness, SEM analysis would offer complementary information regarding grain morphology, film compactness, and cross-sectional structure. Such complementary characterization will be considered in future investigations.

**Figure 4:**
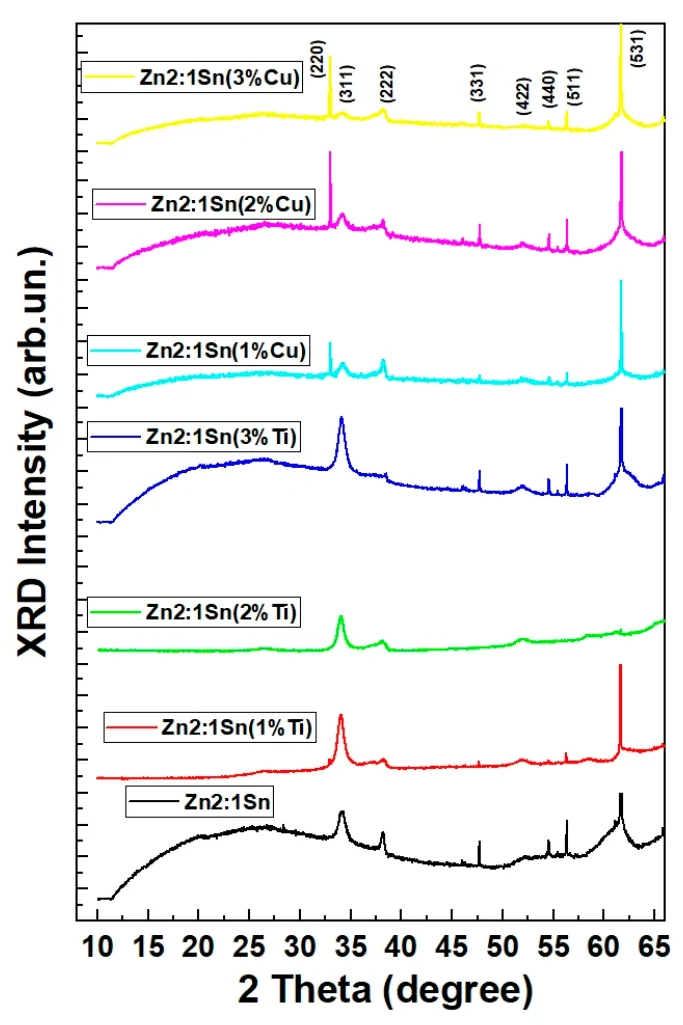
XRD pattern of Ti and Cu doped Zn2:1Sn oxide films obtained by spray pyrolysis.

X-ray diffraction (XRD) patterns of undoped and doped Zn_2_SnO_4_ films deposited by spray pyrolysis are presented in Figure 4. The diffraction patterns indicate that all deposited films exhibit a polycrystalline structure characteristic of cubic spinel Zn_2_SnO_4_. The main diffraction peaks can be indexed to the (220), (311), (222), (331), (422), (440), (511), and (531) crystallographic planes, in agreement with the characteristic reflections of the spinel Zn_2_SnO_4_ phase. The strongest diffraction peak corresponds to the (311) plane, indicating that this crystallographic plane is the dominant diffraction orientation in the deposited films, consistent with previously reported spray-pyrolyzed Zn_2_SnO_4_ films.

The undoped Zn_2_SnO_4_ film exhibits relatively broad diffraction peaks with a pronounced amorphous background, suggesting limited crystallinity. Upon doping with Ti and Cu, a noticeable improvement in structural ordering is observed, as evidenced by the increased intensity and reduced width of the diffraction peaks. This behavior suggests improved structural ordering and a possible increase in coherent crystallite size.

The XRD patterns reveal variations in the intensity and width of the diffraction peaks depending on the type and concentration of the dopant. These changes indicate that variations in the Ti and Cu concentrations are associated with modifications of the structural characteristics of the Zn_2_SnO_4_ films, with distinct variations observed at dopant concentrations of 2–3 vol.%. In the Ti-doped films, crystallinity gradually improves with increasing dopant concentration, although the enhancement is less pronounced than that observed for Cu doping.

No additional diffraction peaks corresponding to crystalline secondary phases were detected within the sensitivity limits of the XRD measurements. This indicates that no detectable crystalline phase separation or structural transformation of the Zn_2_SnO_4_ phase occurs within the investigated range of nominal Ti and Cu concentrations. However, the absence of additional diffraction peaks does not by itself provide definitive evidence for the incorporation of Ti or Cu into the Zn_2_SnO_4_ lattice, particularly at relatively low dopant concentrations or for species present in poorly crystalline or amorphous states.

Overall, the XRD analysis indicates that variations in the nominal Ti and Cu concentrations are accompanied by changes in the crystallinity and structural ordering of the spray-pyrolyzed Zn_2_SnO_4_ films, while the cubic spinel Zn_2_SnO_4_ phase remains the predominant crystalline phase. The improved structural ordering may facilitate charge transport by reducing structural disorder and scattering at grain boundaries; however, the final electrical and photoelectrical behavior also depends on the defects introduced by the dopants.

The present observations are in good agreement with previous reports on spray-pyrolyzed Zn_2_SnO_4_ films, which also describe the predominance of the (311) reflection and the preservation of the cubic spinel structure after moderate Ti or Cu doping [29,30,31].

The structural and morphological characteristics discussed above provide an important basis for understanding the electrical and UV photodetection behavior of the Zn_2_SnO_4_ films. Although Ti and Cu doping preserve the cubic spinel structure, they produce different concentration-dependent modifications of the film morphology and surface characteristics. These differences are accompanied by distinct electrical transport behavior, as reflected by the sheet resistance and current–voltage characteristics, and consequently by different UV photoresponse characteristics. Therefore, the structural and morphological results are not considered independent characterization data here, but part of the structure–property relationship governing the photodetection performance of the films. The electrical and photoelectrical properties are discussed below in relation to these dopant-dependent structural and morphological features.

**Figure 5:**
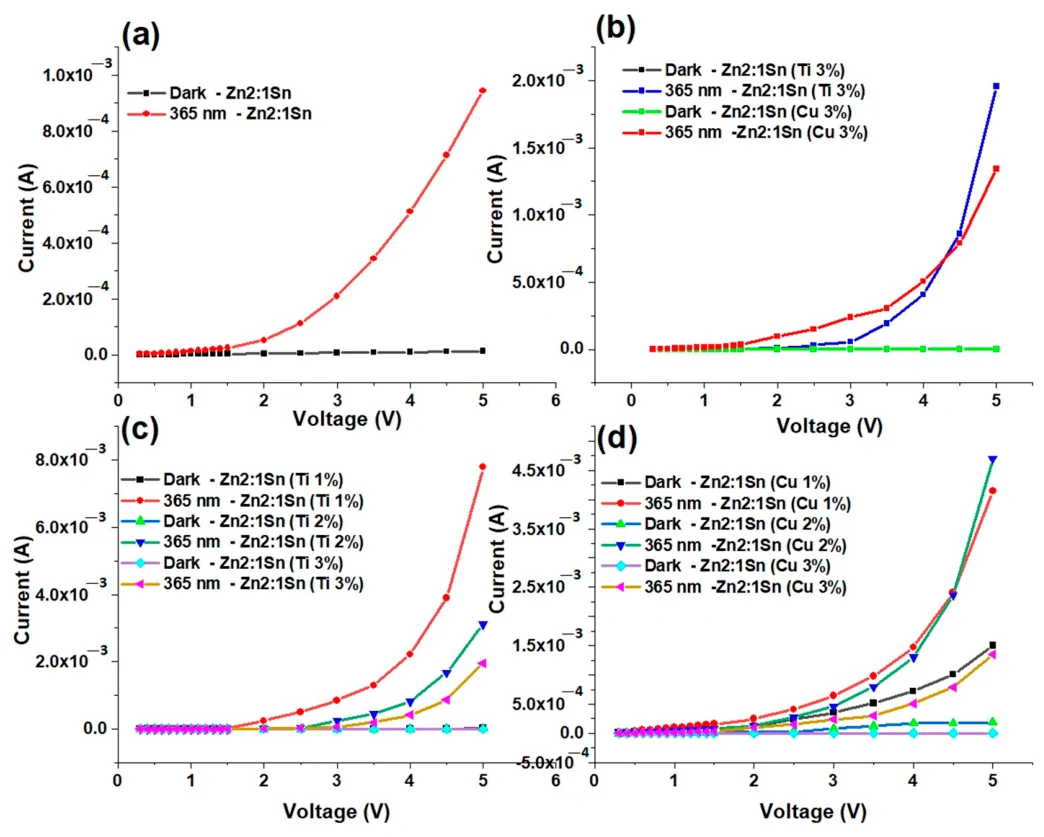
Current–voltage (I–V) characteristics: (**a**) undoped Zn2:1Sn; (**b**) comparison between Zn2:1Sn (Ti 3 vol.%) and Zn2:1Sn (Cu 3 vol.%); (**c**) Ti-doped (1–3 vol.%) Zn2:1Sn films; and (**d**) Cu-doped (1–3 vol.%) Zn2:1Sn films.

Figure 5 presents the current–voltage (I–V) characteristics of Zn2:1Sn films measured under dark conditions and under 365 nm UV illumination for the undoped sample and films doped with Ti and Cu. In all investigated samples, the current exhibits a non-linear dependence on the applied voltage, indicating deviation from purely ohmic conduction. Such behavior may be associated with interfacial barriers and/or defect-related transport processes; however, these contributions cannot be distinguished from the present I–V measurements alone. Upon 365 nm illumination, all samples exhibit a pronounced increase in current, confirming efficient photogeneration of charge carriers and the photoactive nature of the Zn_2_SnO_4_ films.

Figure 5a compares the undoped Zn2:1Sn oxide film under dark and illuminated conditions. The dark current remains very low over the entire voltage range, whereas the photocurrent increases rapidly with increasing bias, reaching approximately 9.5 × 10^−4^ A at 5 V. This large difference between the dark and illuminated currents demonstrates the efficient photogeneration of charge carriers and their effective contribution to electrical conduction under an applied electric field.

Figure 5b compares the photoelectrical performance of Zn2:1Sn oxide films doped with 3 vol.% Ti and 3 vol.% Cu. Both dopants improve the photocurrent under UV illumination; however, the Ti-doped film exhibits the highest photocurrent, approaching 2.0 × 10^−3^ A at 5 V, while the Cu-doped film reaches approximately 1.3 × 10^−3^ A. In contrast, the low dark current indicates a high photo-to-dark current contrast, which is beneficial for UV photodetection.

The influence of Ti concentration is illustrated in Figure 5c. Increasing the Ti content strongly modifies the electrical response of the films. The sample doped with 1 vol.% Ti exhibits the highest photocurrent, reaching nearly 8.0 × 10^−3^ A at 5 V. Further increasing the Ti concentration to 2 and 3 vol.% results in a gradual reduction in the photocurrent, although all doped samples maintain very low dark currents. The reduction in photocurrent at higher Ti concentrations may be associated with concentration-dependent changes in charge transport and carrier relaxation. It should be noted, however, that the present experimental results do not allow the specific contribution of dopant-related defect states, carrier trapping/recombination, or changes in carrier transport to be established directly. Therefore, the observed variations in photocurrent, resistance, and recovery time are interpreted primarily in terms of the experimentally determined structural, morphological, and electrical properties, rather than as direct evidence of a specific microscopic mechanism. Defect states and carrier trapping/recombination processes may contribute to the observed photoelectrical behavior, but their specific roles cannot be uniquely distinguished based on the present measurements. Further investigations, including Hall effect measurements to determine carrier concentration and mobility, together with complementary defect-state characterization, would be required to clarify the underlying mechanisms.

Figure 5d illustrates the influence of Cu concentration on the electrical response of Zn2:1Sn oxide films under dark and UV illumination. Unlike the Ti-doped series, the Cu-doped films exhibit a more pronounced concentration-dependent behavior. The highest photocurrent is observed for the 2 vol.% Cu sample, reaching approximately 4.7 × 10^−3^ A at 5 V, followed closely by the 1 vol.% Cu film, whereas the 3 vol.% Cu sample exhibits a noticeable decrease in photocurrent. The decrease in dark current with increasing nominal Cu concentration indicates a modification of the electrical transport behavior. This variation may be associated with changes in carrier concentration and/or transport pathways; however, the specific microscopic mechanisms cannot be determined from the present measurements.

**Figure 6:**
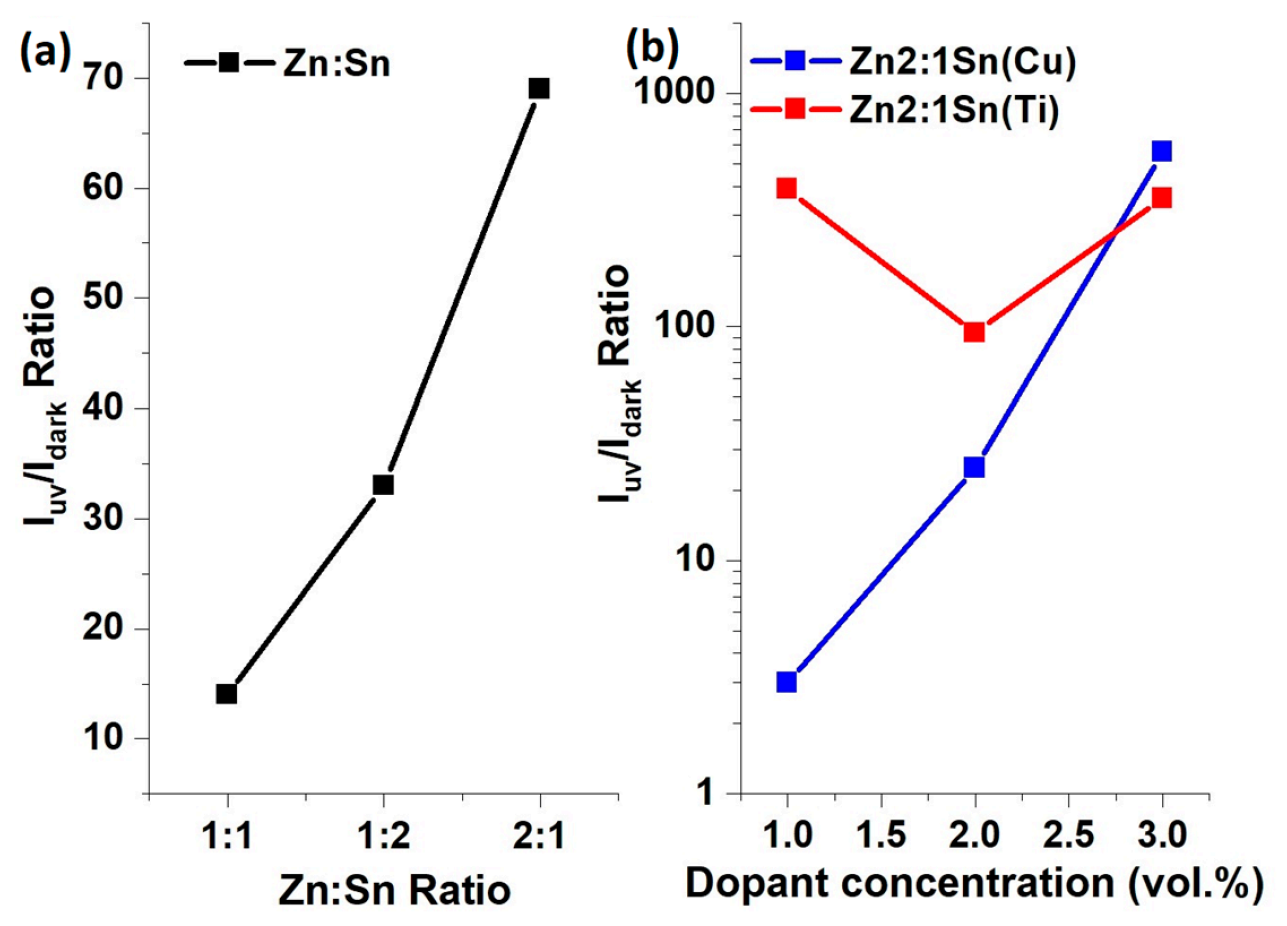
I_uv_/I_dark_ ratio derived from I-V characteristics of (**a**) undoped and (**b**) Ti- and Cu-doped Zn2:1Sn oxide films obtained by spray pyrolysis.

Overall, both Ti and Cu doping substantially improve the UV photoresponse of Zn2:1Sn oxide films. The existence of an optimum dopant concentration indicates that the beneficial effects associated with doping do not continue to increase with dopant concentration. The reduction in photoresponse at higher dopant concentrations may be related to changes in carrier transport, trapping, and relaxation processes; however, the individual contributions of these mechanisms cannot be distinguished from the present measurements. The investigated compositions, the 1 vol.% Ti and 3 vol.% Cu (Figure 6) samples, exhibit the best photoelectrical performance, combining high photocurrent with low dark current, making them promising candidates for UV photodetectors requiring a high photoresponse combined with a low dark current.

The I–V measurements demonstrate that both Ti and Cu doping markedly enhance the UV photoresponse of Zn_2_SnO_4_ films. However, the optimum dopant concentration depends on the dopant species, indicating that Ti and Cu produce different concentration-dependent electrical responses.

To further quantify the photoresponse observed in the I–V characteristics presented in Figure 5, the UV sensing performance was evaluated using the photo-to-dark current ratio (I_UV_/I_dark_), as summarized in Figure 6. This parameter provides a simple figure of merit describing the contrast between the illuminated and dark current, and is commonly used to evaluate the photoresponse of photodetectors.

Figure 6a shows that the I_UV_ /I_dark_ ratio strongly depends on the Zn:Sn precursor ratio. The photoresponse increases progressively from approximately 14 for the Zn:Sn = 1:1 sample to about 33 for the Zn:Sn = 1:2 composition, reaching a maximum value of nearly 67 for the Zn2:1Sn film. This continuous improvement confirms the results obtained from the I–V measurements, where the Zn2:1Sn sample exhibited the highest photocurrent while maintaining a relatively low dark current. The enhanced photoresponse can therefore be attributed to the optimized electrical transport and efficient separation of photogenerated charge carriers in the Zn2:1Sn composition.

Based on these results, the Zn2:1Sn oxide film was selected as the host material for further doping with Ti and Cu. As shown in Figure 6b, the incorporation of both dopants significantly modifies the I_UV_/I_dark_ ratio, although their effects differ considerably. The Ti-doped films exhibit a high photoresponse at 1 vol.% (≈430), followed by a decrease to approximately 96 at 2 vol.% and subsequent recovery to around 326 at 3 vol.%. In contrast, the Cu-doped films show a nearly monotonic increase in the photo-to-dark current ratio, rising from approximately 3 at 1 vol.% to about 25 at 2 vol.% and reaching the highest value of nearly 763 at 3 vol.%.

The distinct evolution of the photo-to-dark current ratio demonstrates that Ti and Cu affect the photoelectrical response of Zn_2_SnO_4_ differently. While Ti doping provides the highest current ratio at 1 vol.%, Cu doping exhibits a continuous improvement with increasing concentration, reaching its maximum at 3 vol.%. These results indicate that the optimum dopant concentration depends on the dopant species and highlight the different roles of Ti and Cu in tailoring the UV sensing performance of Zn_2_SnO_4_ films.

**Figure 7:**
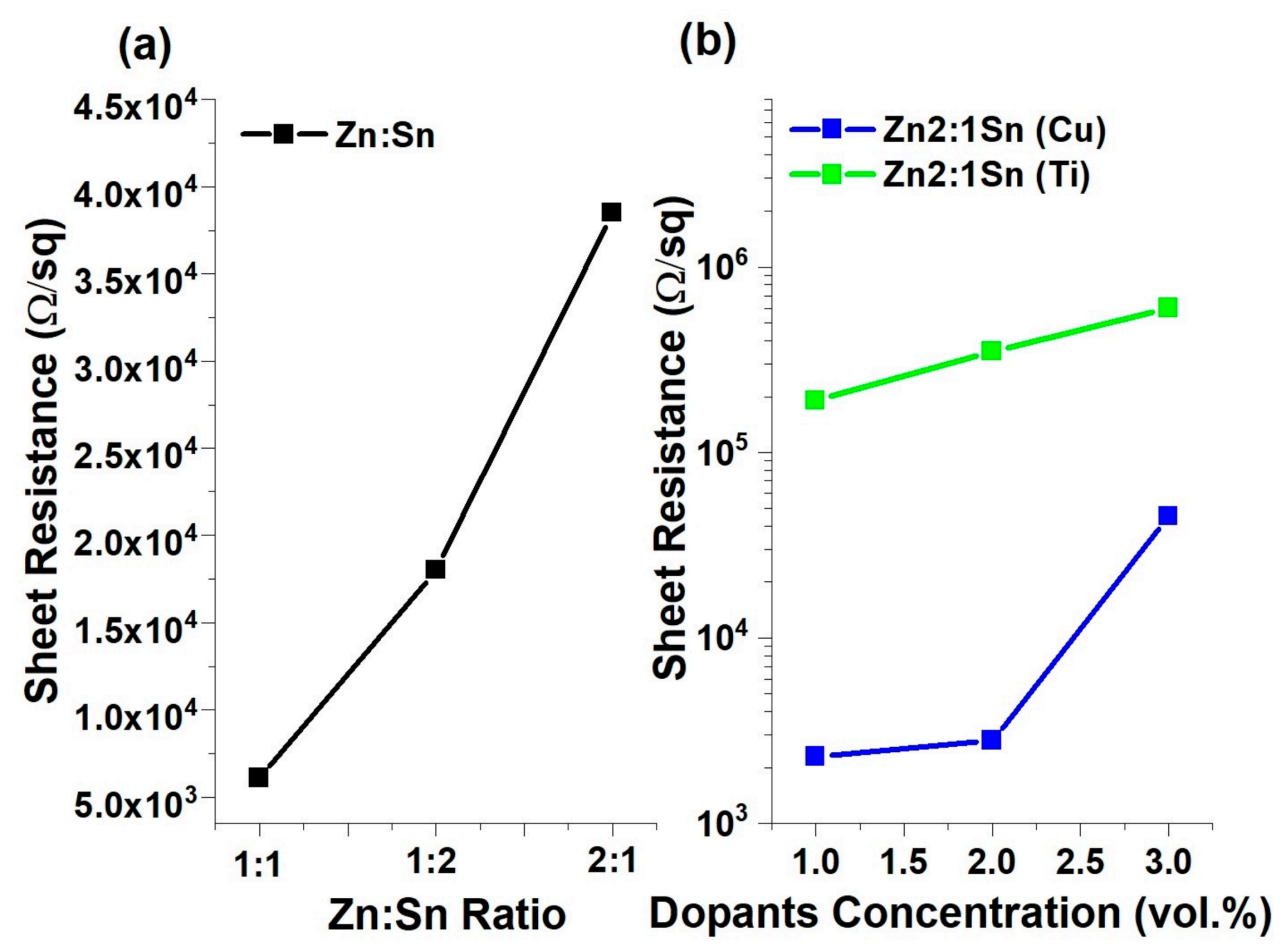
The sheet resistance of ZnSnO films: (**a**) the dependence on the Zn:Sn precursor ratio and (**b**) the dependence on Ti and Cu dopant concentration (1–3 vol.%) for Zn2:1Sn films. Measurements were performed using the four-point probe method. For clarity, the sheet resistance values in (**b**) are presented on a logarithmic scale owing to the large difference between the Ti- and Cu-doped samples.

To better understand the origin of the enhanced photoresponse discussed above, the electrical properties of the films were further investigated by analyzing their sheet resistance, as shown in Figure 7. Since the photo-to-dark current ratio depends on both the photocurrent and the dark current, the sheet resistance provides useful information regarding the electrical transport properties of the sensing layer.

Figure 7a shows the variation in sheet resistance with Zn:Sn precursor ratio. The sheet resistance increases systematically from approximately 6.1 × 10^3^ Ω/sq for the Zn:Sn = 1:1 sample to 1.8 × 10^4^ Ω/sq for the Zn:Sn = 1:2 composition, reaching nearly 4.0 × 10^4^ Ω/sq for the Zn2:1Sn film. This increase in resistance is consistent with the higher I_UV_/I_dark_ ratio observed in Figure 5a, suggesting that the increase in sheet resistance contributes to the reduction in the dark current, thereby improving the photo-to-dark current ratio.

**Figure 8:**
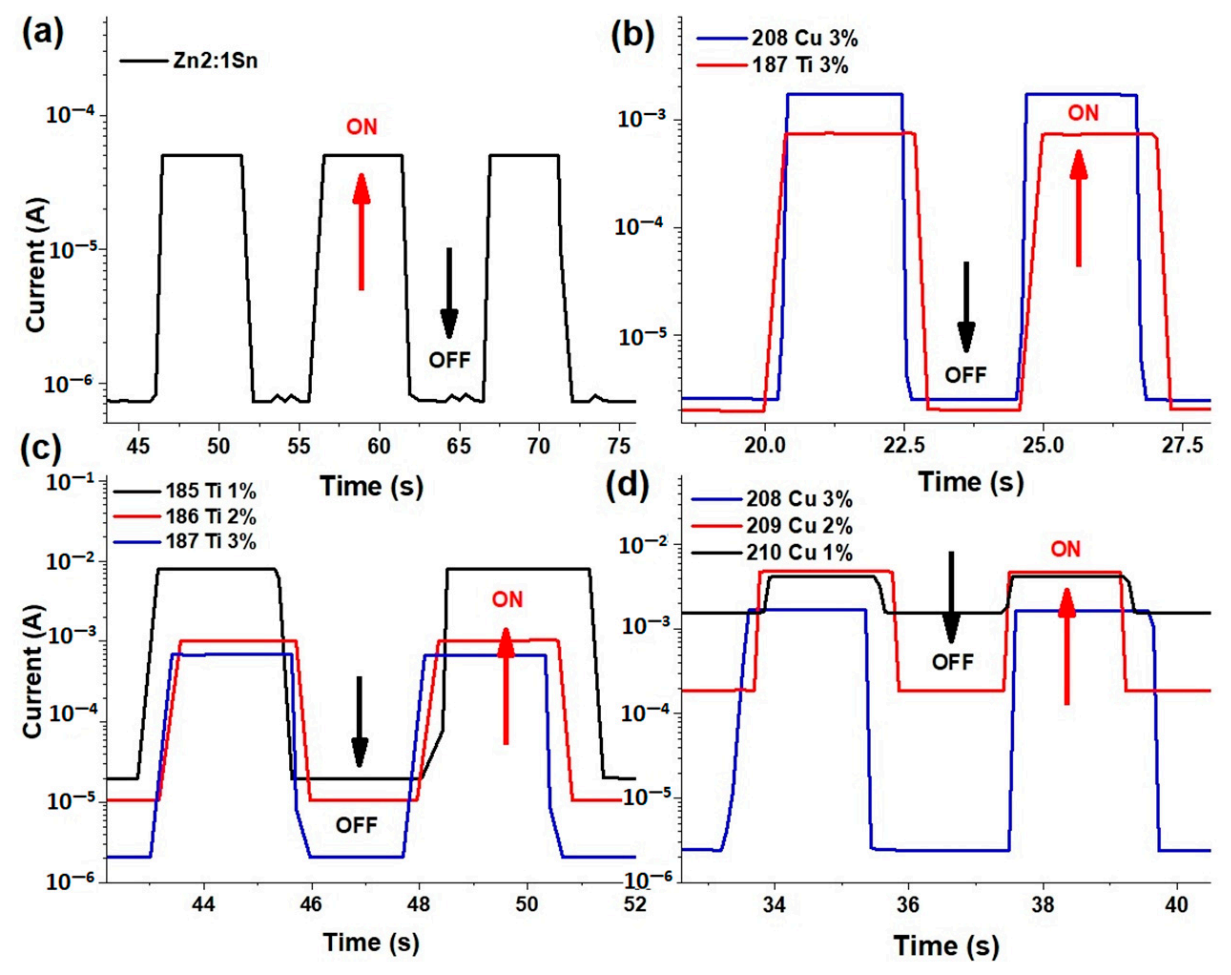
Transient photocurrent (I–t) response of Zn2:1Sn oxide films under periodic UV light ON/OFF switching: (**a**) undoped Zn2:1Sn; (**b**) comparison between Zn2:1Sn (Ti 3 vol.%) and Zn2:1Sn (Cu 3 vol.%); (**c**) Ti-doped (1–3 vol.%) Zn2:1Sn films; and (**d**) Cu-doped (1–3 vol.%) Zn2:1Sn films.

The effect of dopant concentration on the sheet resistance of Zn2:1Sn oxide films is presented in Figure 7b. A remarkable difference is observed between Ti- and Cu-doped films. Throughout the investigated concentration range, Ti-doped films exhibit sheet resistance values approximately one to two orders of magnitude higher than those of the Cu-doped films. Ti doping results in a gradual increase in sheet resistance from approximately 1.9 × 10^5^ Ω/sq as the concentration increases from 1 to 3 vol.%. In contrast, Cu-doped films exhibit considerably lower sheet resistance, remaining close to 2–3 × 10^3^ Ω/sq for 1–2 vol.% Cu before increasing to approximately 4.5 × 10^4^ Ω/sq at 3 vol.%. These results indicate that sheet resistance alone does not determine the UV sensing performance. Although higher resistance can contribute to dark current suppression, the resulting I_UV_/I_dark_ ratio depends on the balance between reduced dark current and preservation of a sufficiently high photocurrent. This is particularly evident for the Ti-doped films, which exhibit consistently high sheet resistance but a non-monotonic variation in the photo-to-dark current ratio. In contrast, for the 3 vol.% Cu film, the pronounced increase in sheet resistance coincides with strong dark current suppression while a measurable photocurrent is retained, resulting in the highest I_UV_/I_dark_ ratio. These findings demonstrate that compositional engineering provides an effective approach for tailoring the electrical transport properties and, consequently, optimizing the UV sensing performance of Zn_2_SnO_4_ films. The photoresponse characteristics of the undoped and doped Zn2:1Sn oxide films were evaluated by monitoring the time-dependent photocurrent under periodic UV light irradiation. Figure 8 presents the transient photoresponses recorded during repeated UV ON/OFF switching cycles, providing information on the repeatability, operational stability, and switching behavior of the fabricated photodetectors.

As shown in Figure 8a, the undoped Zn2:1Sn oxide film exhibits a stable and reproducible transient photocurrent response. The current increases rapidly upon UV illumination and returns to its initial level after the light is switched off, with nearly identical photocurrent amplitudes observed during consecutive ON/OFF cycles. This behavior confirms the good operational stability and reversible photoresponse of the undoped film.

Figure 8b compares the transient photocurrent responses of the Zn2:1Sn oxide films doped with 3 vol.% Ti and 3 vol.% Cu. Both samples exhibit stable and reproducible switching behavior with well-defined ON and OFF states, confirming the reliability of the fabricated photodetectors. Under identical measurement conditions, the Cu-doped film produces a considerably higher photocurrent than the corresponding Ti-doped film, demonstrating its superior photoresponse. The influence of Ti concentration on the transient photocurrent response is presented in Figure 8c. Increasing the Ti content from 1 to 3 vol.% results in a gradual decrease in the photocurrent amplitude, while the dark current also decreases. Despite these changes, all Ti-doped samples retain stable and reproducible ON/OFF switching characteristics, indicating that Ti incorporation does not compromise the operational stability of the photodetectors.

Figure 8d illustrates the effect of Cu concentration on the transient photocurrent response. The photocurrent increases with increasing Cu concentration from 1 to 3 vol.% Cu, reaching its maximum at 3 vol.% Cu, with a photocurrent-to-dark current ratio of 763. All Cu-doped samples exhibit stable and reproducible switching characteristics, with sharp transitions between the illuminated and dark states. However, further increasing the Cu concentration does not lead to an additional improvement in the transient photoresponse, while the excellent switching stability is preserved. Overall, the transient photocurrent measurements demonstrate that both Ti and Cu doping preserve the stable switching behavior of Zn2:1Sn oxide photodetectors. While Ti and Cu modify the photocurrent amplitude differently, neither dopant adversely affects the repeatability or reversibility of the photoresponse. These results confirm that the photoelectrical performance of Zn2:1Sn oxide films can be effectively tailored through controlled doping while maintaining stable device operation.

**Figure 9:**
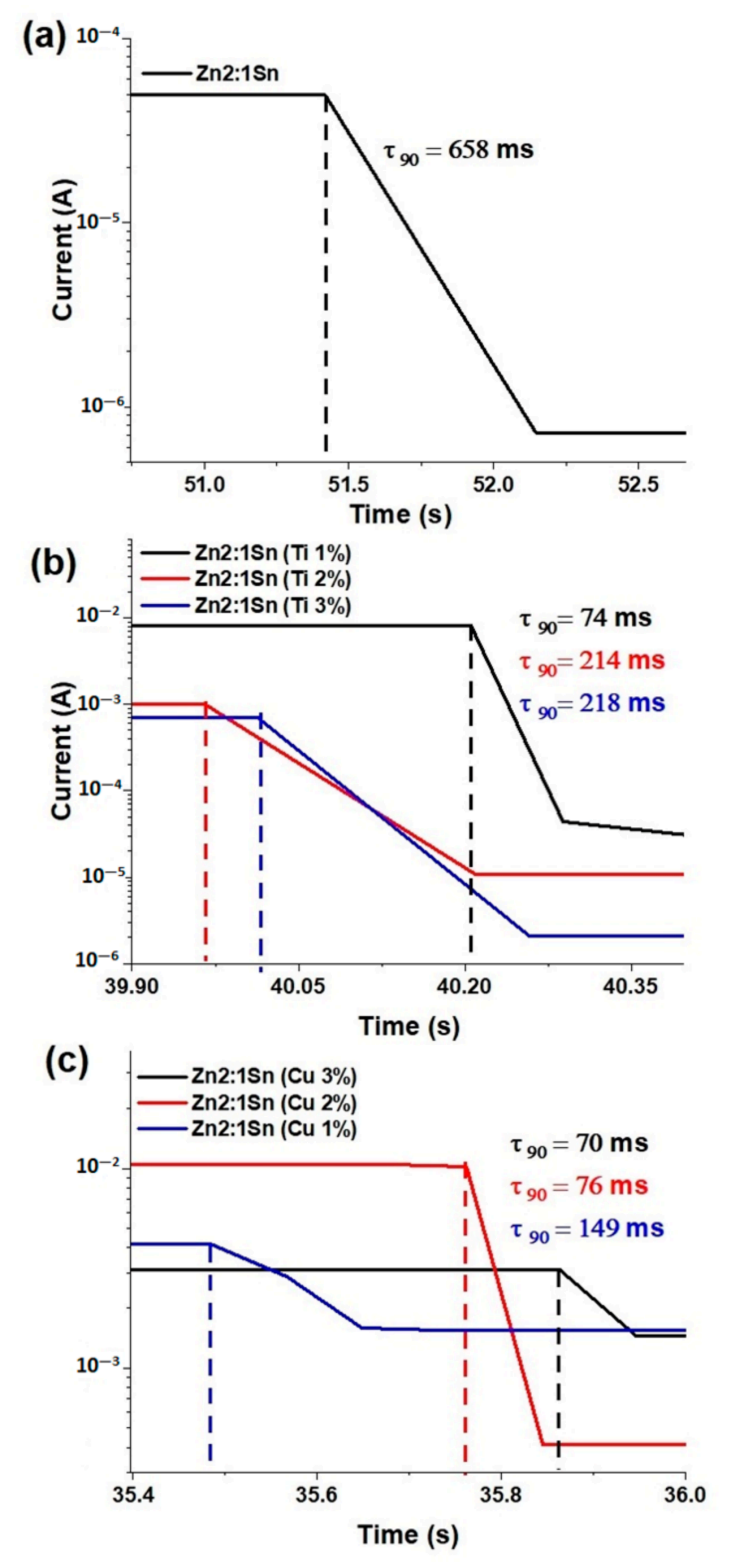
Recovery time analysis of the transient photocurrent response of Zn2:1Sn oxide film photodetectors under UV illumination: (**a**) undoped Zn2:1Sn film; (**b**) Ti-doped Zn2:1Sn films with Ti concentrations of 1–3 vol.%; and (**c**) Cu-doped Zn2:1Sn films with Cu concentrations of 1–3 vol.%. The recovery time (τ_90_) was determined from the transient photocurrent curves after switching the UV illumination from ON to OFF.

The response dynamics of the obtained Zn2:1Sn oxide photodetectors were further evaluated by analyzing the recovery process after switching off the UV illumination. Figure 9 presents the recovery times (τ_90_) of the undoped and Ti- and Cu-doped Zn_2_SnO_4_ films, together with the corresponding recovery times (τ_90_), providing insight into the carrier relaxation dynamics following the removal of UV illumination. After switching off the UV illumination, the photocurrent decreases and subsequently remains nearly constant.

The recovery time (τ_90_) was determined using a 90% recovery criterion. The τ_90_ value was defined as the time required for 90% of the photocurrent variation to be recovered after switching off the illumination. This criterion provides a consistent and reproducible method for comparing the recovery dynamics of the undoped and Ti- and Cu-doped Zn_2_SnO_4_ photodetectors.

As shown in Figure 9a, the undoped Zn2:1Sn oxide film exhibits a recovery time of approximately 658 ms, indicating a relatively slow recovery time of the photocurrent after the UV illumination is switched off. The prolonged recovery suggests the presence of slow carrier relaxation processes, which delay return of the current to its initial dark value.

The influence of Ti doping on the recovery characteristics is presented in Figure 9b. A significant reduction in the recovery time is observed after Ti incorporation. The Zn2:1Sn oxide films doped with 1, 2, and 3 vol.% Ti exhibit recovery times of 74, 214, and 218 ms, respectively. Among the investigated Ti-doped samples, the 1 vol.% Ti film demonstrates the fastest recovery, whereas higher Ti concentrations are associated with a slower recovery process, indicating that the beneficial effect of Ti incorporation is maximized at low dopant concentrations. Figure 9c illustrates the effect of Cu doping on the recovery behavior. The recovery times (τ_90_) are 149 ms for 1 vol.% Cu, 76 ms for 2 vol.% Cu, and 70 ms for 3 vol.% Cu, indicating a substantial improvement compared with the undoped film. The shortest recovery times (τ_90_) indicate faster relaxation of the photoinduced current and may be associated with changes in carrier transport and relaxation dynamics induced by Cu doping. However, the present measurements do not directly distinguish the individual contributions of carrier recombination, trapping, and transport to the observed recovery behavior. The sharp recovery time after switching off the UV illumination further demonstrates the excellent switching characteristics of the Cu-doped photodetectors. Overall, both Ti and Cu doping significantly accelerate the recovery process compared with the undoped Zn2:1Sn film. The 1 vol.% Ti film exhibits the fastest response within the Ti-doped series. These results demonstrate that the response dynamics can be effectively tailored through appropriate dopant selection and concentration, highlighting the potential of both Ti and Cu doping for improving the switching performance of Zn2:1Sn oxide UV photodetectors. It is noteworthy that the optimum composition depends on the selected performance criterion. While the 1 vol.% Ti-doped film exhibits the highest photocurrent together with the fastest recovery within the Ti-doped series, the 3 vol.% Cu-doped film combines one of the shortest recovery times (τ_90_) of 70 ms with the highest photo-to-dark current ratio (763), demonstrating the complementary advantages of the two doping strategies.

**Figure 10:**
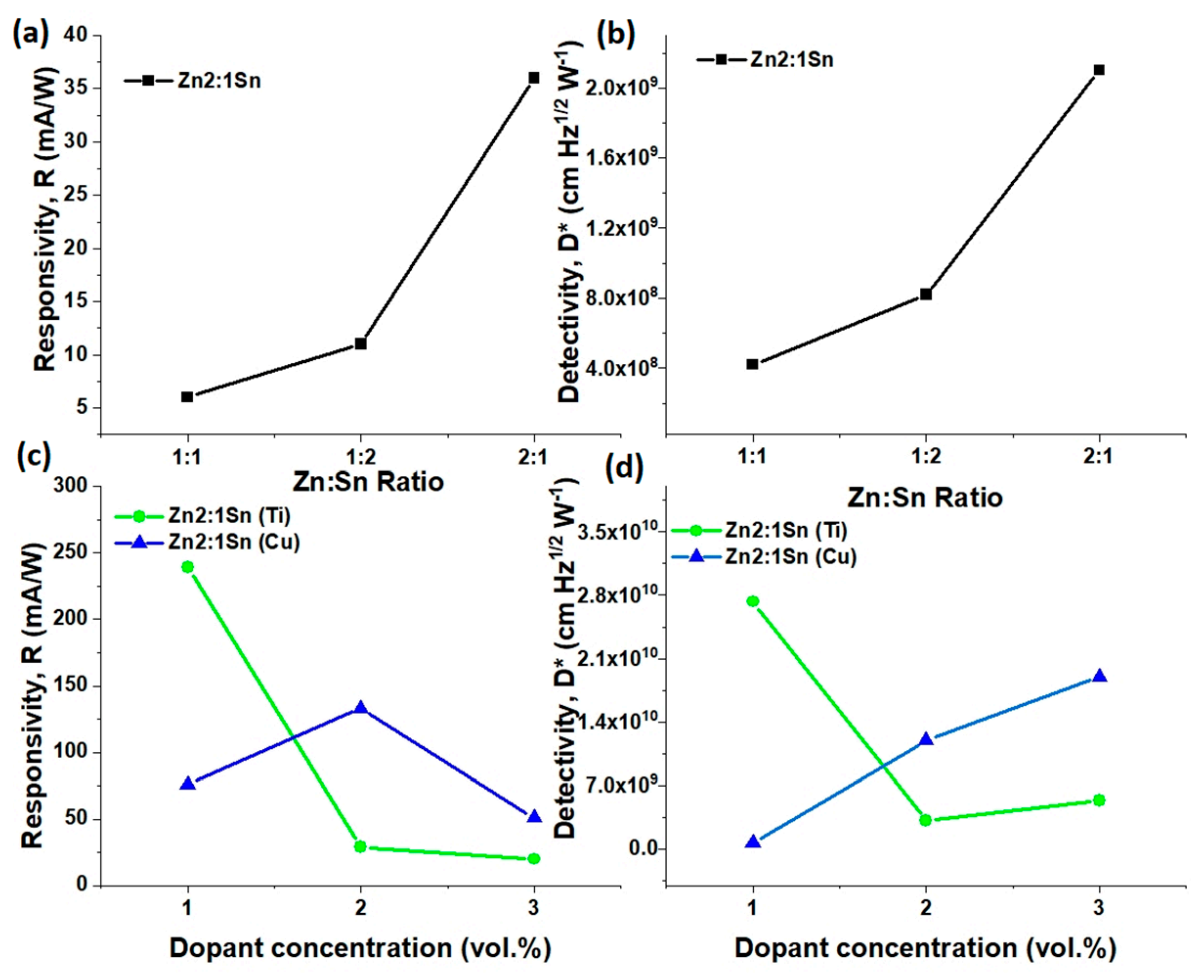
The dependence of the photoelectrical performance of ZnSnO film photodetectors on the Zn:Sn precursor ratio and dopant concentration: (**a**) the responsivity (R) and (**b**) specific detectivity (D*) of undoped Zn2:1Sn films with different Zn:Sn precursor ratios (1:1, 1:2, and 2:1); (**c**) the responsivity (R) and (**d**) specific detectivity (D*) of Zn2:1Sn films doped with Ti and Cu (1–3 vol.%).

To evaluate the influence of the Zn:Sn precursor ratio and dopant concentration on the photoelectrical performance of the fabricated photodetectors, the responsivity (R) and specific detectivity (D*) were calculated from the illuminated and dark currents obtained from the I–V characteristics at an applied bias voltage of 5 V, using an effective active area of 0.08 cm^2^ and an incident optical power of approximately 0.0344 W following the equations in (1):

(1) $$R=\frac{I_{UV}-I_{dark}}{P_{ill}},\;D^{*}=\frac{R\sqrt{A}}{\sqrt{2eI_{dark}}},$$

where I_UV_ is the photocurrent of the photodetector under UV illumination, I_dark_ is the dark current, P_ill_ is the incident optical power, A is the active area of the photodetector, and e is the elementary charge [32]. Figure 10a,b illustrate the dependence of the responsivity and detectivity on the Zn:Sn precursor ratio for the undoped ZnSnO films. Both parameters increase systematically with increasing Zn content, indicating a significant improvement in the photoresponse of the photodetectors. The Zn2:1Sn oxide film exhibits the highest responsivity (36 mA/W) and detectivity (2.1 × 10^9^ Jones) among the investigated undoped films, demonstrating the superior photoelectrical performance of the Zn-rich composition. Consequently, this composition was selected as the reference material for subsequent Ti and Cu doping studies.

The effect of dopant concentration on the photoelectrical performance of Zn2:1Sn oxide films is presented in Figure 10c,d. For Ti-doped samples, the responsivity decreases continuously with increasing Ti concentration from 239 to 20 mA/W, whereas the detectivity initially decreases from 2.73 × 10^10^ Jones at 1 vol.% Ti to 3.2 × 10^9^ Jones at 2 vol.% Ti, followed by partial recovery to 5.4 × 10^9^ Jones at 3 vol.% Ti. This behavior indicates that increasing the Ti concentration progressively reduces the responsivity, while the partial recovery of detectivity at 3 vol.% reflects the simultaneous reduction in the dark current.

In contrast, Cu doping exhibits a different trend. The responsivity increases from 1 to 2 vol.% Cu (from 76 to 133 mA/W) and subsequently decreases at 3 vol.% Cu (51 mA/W), indicating the existence of an optimum Cu concentration for efficient photocarrier generation. Meanwhile, the detectivity increases monotonically with Cu concentration, reaching its maximum value for the 3 vol.% Cu-doped film (1.9 × 10^10^ Jones). Unlike responsivity, which reaches its maximum at 2 vol.% Cu, the detectivity increases continuously with increasing Cu concentration, reaching its highest value at 3 vol.% Cu. This observation highlights that maximizing the photocurrent does not necessarily lead to the highest detectivity, as the latter is also strongly influenced by the dark current. The enhanced detectivity is mainly associated with the simultaneous increase in photocurrent and suppression of the dark current, leading to an improved signal-to-noise ratio.

These results demonstrate that the photoelectrical performance of Zn_2_SnO_4_ photodetectors strongly depends on both the Zn:Sn precursor ratio and the dopant species. While the Zn2:1Sn composition exhibits the best performance among the undoped films, Ti and Cu doping optimize different performance parameters. Ti doping provides the highest responsivity at low dopant concentration. Within the Cu-doped series, the specific detectivity increases with increasing Cu concentration. This distinction highlights that the optimum dopant concentration depends on the performance criterion considered.

A summary of all the main photoresponse parameters, including the photo-to-dark current ratio, responsivity (R), detectivity (D), and recovery time (τ_90_), is presented in Table 2.

The distinct electrical and photoelectrical responses indicate that Ti and Cu affect the charge transport behavior of Zn_2_SnO_4_ in different ways. Ti-doped films exhibit substantially higher sheet resistance and a relatively stable surface morphology, whereas Cu doping produces a stronger concentration-dependent morphological evolution and considerably lower sheet resistance at 1–2 vol.% Cu. These differences are accompanied by distinct trends in photocurrent, dark current, photo-to-dark current ratio, responsivity, and recovery time. Thus, the enhancement in UV photodetection cannot be attributed solely to an increase in photocurrent. Depending on the dopant and its concentration, the overall response is also strongly influenced by dark current suppression and the resulting charge transport and relaxation behavior. Although the present measurements do not directly identify specific dopant-related defect states or substitutional sites, the observed concentration-dependent trends provide experimental evidence that Ti and Cu modify the electrical and photoelectrical behavior of Zn_2_SnO_4_ through different pathways.

## 4. Conclusions

Zn2:1Sn oxide films doped with Ti (1–3 vol.%) and Cu (1–3 vol.%) were successfully deposited by spray pyrolysis, and the influence of dopant type and concentration on their structural, morphological, electrical, and UV photodetection properties was systematically investigated.

XRD analysis confirmed the preservation of the cubic spinel Zn_2_SnO_4_ structure after Ti and Cu doping, with no detectable crystalline secondary phases. Doping also improved the structural ordering of the films, with the effect being more pronounced for Cu than for Ti. AFM analysis revealed that Ti doping maintained a relatively homogeneous surface morphology, whereas Cu induced a concentration-dependent morphological evolution, with maximum surface roughness observed at 2 vol.% Cu followed by partial surface homogenization at 3 vol.% Cu. All doped films exhibited an enhanced UV photoresponse compared with the undoped sample. The maximum photocurrent was obtained for the 1 vol.% Ti- and 2 vol.% Cu-doped films, whereas the 3 vol.% Cu-doped film exhibited the highest photo-to-dark current ratio (≈763). In contrast, the Zn2:1Sn (Ti 1 vol.%) photodetector demonstrated the best detection performance, with a responsivity and specific detectivity reaching approximately 239 mA/W and 2.7 × 10^10^ Jones, respectively. Transient measurements demonstrated a consistent ON/OFF switching response over two consecutive illumination cycles for all samples, with Cu-doped films exhibiting the fastest recovery times (τ_90_), approximately 70–149 ms, representing an approximately ninefold improvement over the undoped film.

The results demonstrate that Ti and Cu doping produce distinctly different structural, morphological, electrical, and UV photodetection responses in Zn_2_SnO_4_ thin films. Ti doping maintains a relatively homogeneous surface morphology and favors high sheet resistance, with 1 vol.% Ti providing the highest responsivity and specific detectivity. In contrast, Cu doping induces stronger concentration-dependent morphological changes and substantially lower sheet resistance at 1–2 vol.% Cu, while 3 vol.% Cu provides a high photo-to-dark current ratio (763) and rapid recovery time (τ_90_) of 70 ms.

The novelty of this work lies in the systematic and direct comparison of Ti- and Cu-doped Zn_2_SnO_4_ thin films prepared under identical spray pyrolysis and measurement conditions. This approach enables the dopant-dependent relationships between morphology, electrical transport, and UV photodetection performance to be directly evaluated. The results demonstrate that Ti and Cu provide complementary routes for tailoring Zn_2_SnO_4_-based UV photodetectors: Ti doping favors high responsivity and specific detectivity, whereas Cu doping, particularly at 3 vol.%, promotes a high photo-to-dark current ratio and fast recovery time. Thus, the choice and concentration of the dopant can be used to selectively tailor the performance characteristics of Zn_2_SnO_4_ UV photodetectors.

## Figures and Tables

**Table 1:** Surface roughness parameters and grain size distribution of undoped and Ti- and Cu-doped Zn2:1Sn oxide films.

	Particle Statistics		Surface Morphology	
Samples	Mean Particle Size μ (nm)	Standard Deviation σ (nm)	RMS Roughness Sq, (nm)	Mean Roughness Sa, (nm)
Zn2:1Sn (Ti 1 vol.%)	92.3	18.5	4.425	3.164
Zn2:1Sn (Ti 2 vol.%)	69.5	14.1	4.287	3.386
Zn2:1Sn (Ti 3 vol.%)	79.1	15.7	5.130	4.125
Zn2:1Sn (Cu 1 vol.%)	64.4	12.7	6.051	4.511
Zn2:1Sn (Cu 2 vol.%)	118.1	24.9	21.56	16.24
Zn2:1Sn (Cu 3 vol.%)	85.7	18.5	6.022	4.767
Zn2:1Sn	98.8	17.9	15.76	10.40

**Table 2:** Photoresponse characteristics of Zn2:1Sn oxide films doped with Ti and Cu.

Samples	Photo-to Dark Current Ratio	Responsivity R, (mA/W)	Detectivity, D* (cm Hz^1/2^W^−1^)	Recovery Time, τ_90_ (ms)
Zn2:1Sn (Ti 1 vol.%)	430	239	2.73·10^10^	74
Zn2:1Sn (Ti 2 vol.%)	96	29	3.2·10^9^	214
Zn2:1Sn (Ti 3vol.%)	326	20	5.4·10^9^	218
Zn2:1Sn (Cu 1vol.%)	3	76	7.3·10^8^	149
Zn2:1Sn (Cu 2 vol.%)	25	133	1.2·10^10^	76
Zn2:1Sn (Cu 3 vol.%)	763	51	1.9·10^10^	70
Zn2:1Sn	67	36	2.1·10^9^	658

## Data Availability

The original contributions presented in this study are included in the article. Further inquiries can be directed to the corresponding author.
